# Ten years of surveillance of the Yulong plague focus in China and the molecular typing and source tracing of the isolates

**DOI:** 10.1371/journal.pntd.0006352

**Published:** 2018-03-30

**Authors:** Peng Wang, Liyuan Shi, Fuxin Zhang, Ying Guo, Zhikai Zhang, Hongli Tan, Zhigang Cui, Yibo Ding, Ying Liang, Yun Liang, Dongzheng Yu, Jianguo Xu, Wei Li, Zhizhong Song

**Affiliations:** 1 Yunnan Provincial Key Laboratory for Zoonosis Control and Prevention, Yunnan Institute for Endemic Disease Control and Prevention, Dali city of Yunnan province, China; 2 Lijiang Center for Disease Control and Prevention, Lijiang City of Yunnan province, China; 3 State Key Laboratory for Infectious Disease Prevention and Control, National Institute for Communicable Disease Control and Prevention, China CDC, Changping, Beijing, China; 4 Yunnan Center for Disease Control and Prevention, Kunming City of Yunnan province, China; Beijing Institute of Microbiology and Epidemiology, CHINA

## Abstract

Plague, caused by *Yersinia pestis*, was classified as a reemerging infectious disease by the World Health Organization. The five human pneumonic plague cases in Yulong County in 2005 gave rise to the discovery of a Yulong plague focus in Yunnan province, China. Thereafter, continuous wild rodent plague (sylvatic plague) was identified as the main plague reservoir of this focus. In this study, the epizootics in Yulong focus were described, and three molecular typing methods, including the different region (DFR) analysis, clustered regularly interspaced short palindromic repeats (CRISPRs), and the multiple-locus variable number of tandem repeats (VNTR) analysis (MLVA) (14+12), were used for the molecular typing and source tracing of *Y*. *pestis* isolates in the Yulong plague focus. Simultaneously, several isolates from the vicinity of Yunnan were used as controls. The results showed that during the 10-year period from 2006 to 2016, an animal plague epidemic occurred in 6 of those years, and 5 villages underwent an animal plague epidemic within a 30-km^2^ area of the Yulong plague focus. Searching for dead mice was the most effective monitoring method in this plague focus. No positive sample has been found in 6937 captured live rodents thus far, suggesting that the virulence of strains in the Yulong plague focus is stronger and the survival time of mice is shorter after infection. Strains from Lijiang, Sichuan and Tibet were of the same complex based on a typing analysis of DFR and CRISPR. The genetic relationship of *Y*. *pestis* illustrated by MLVA “14+12” demonstrates that Tibet and Sichuan strains evolved from the strains 1.IN2 (Qinghai, 1970 and Tibet, 1976), and Lijiang strains are closer to Batang strains (Batang County in Sichuan province, 2011, Himalaya marmot plague foci) in terms of genetic or phylogenic relationships. In conclusion, we have a deeper understanding of this new plague focus throughout this study, which provides a basis for effective prevention and control.

## Author summary

Plague is a type of zoonosis that is highly lethal to humans. The surveillance of animal hosts is critical for the prevention and control of plague. The Yulong plague focus is a newly discovered plague focus in China in recent years. The plague outbreak had attracted widespread attention because 5 people were infected in 2005, 2 of whom died. We have monitored the plague focus for a decade, and isolated strains and DNAs of *Yersinia pestis* were studied. The structure, origin and evolutionary trend of the Yulong plague focus were clarified, which provides a scientific basis for the effective prevention and control of human plague. This article also provides a set of paradigms for the systematic study of new plague foci, which is a perfect combination of traditional monitoring methods and modern research methods.

## Introduction

Plague is an acute infectious disease caused by *Yersinia pestis* (*Y*. *pestis*). Four *Y*. *pestis* biovars have been recognized based on their biochemical properties, i.e., *Antiqua*, *Mediaevalis*, *Orientalis* and *Microtus*. Each *Y*. *pestis* biovar has a different geographic distribution throughout the world [1]. Three devastating plague pandemics have occurred in the last 1500 years worldwide. The third plague pandemic, caused by *Y*. *pestis Orientalis*, originated in the Yunnan province of China in the middle of 19th century and eventually affected more than 60 countries and regions in Asia, Europe, America and Africa [2]. The population structure of *Y*. *pestis* as a clonal lineage with five branches designated 0, 1, 2, 3 and 4. The *Y*. *pestis* genealogy is rooted by *Y*. *pseudotuberculosis* at the base of branch 0, and SNPs have accumulated serially along branch 0 and subsequently along branches1, 2, 3 and 4. There are nine branching lineages (0.ANT1, 0.PE7, 0.ANT3, 0.ANT2, 0.PE2, 0.PE3, 0.PE4A, 0.PE4B and 0.PE4C) in branch 0, seven (1.IN1, 1.ORI1, 1.ORI3, 1.IN3, 1.IN2, 1.ANT and 1.ORI2) in branch 1, two (3.ANT1 and 3.ANT2) in branch 3 and only one (4.ANT1) in branch 4[3].

**Fig 1:**
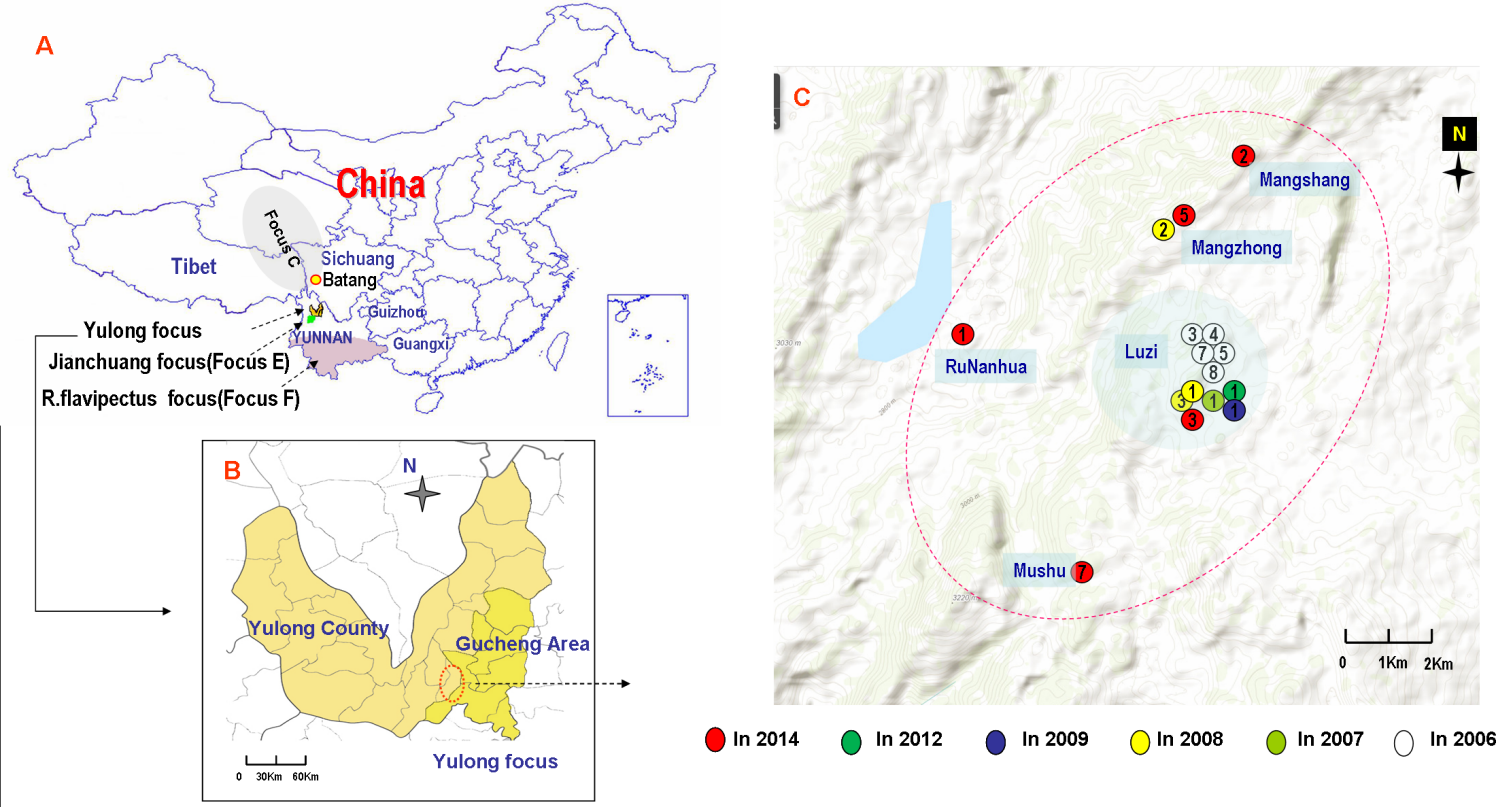
Distribution of sylvatic plagues in the Yulong plague focus. A: Geographic location of three plague foci in Yunnan province, China. B and C: Distribution of sylvatic plagues in the Yulong Plague Focus (2006-2016).

Yunnan province is located in southwestern China and borders with Burma, Laos, and Vietnam. It is adjacent to Guizhou, Guangxi, and Sichuan provinces and Tibet in China. Three plague foci exist in Yunnan (Reference the map in Fig 1): the *Rattus flavipectus* plague focus (Biovar *Orientalis* and genealogy 1.ORI2, termed as focus F in studies [4–8]; termed as focus A in study [9]), the Jianchuan plague focus (Biovar *Antique* and genealogy 1.IN3, focus E in studies [4–8]; termed as focus B in study [9]), and the Yulong plague focus (termed as focus P in reference study [4]). The discovery of the Yulong focus originated from a human plague outbreak (five pneumonia plague cases with two deaths) in Luzi valley of Yulong county in 2005 [4, 9, 10]. Active animal surveillance has been conducted annually in Yulong and neighboring areas since the focus was discovered. In fact, both the Yulong plague focus and the Jianchuan plague focus are located in the middle part of the Hengduan Mountains, and the two foci are adjacent to one another, with similar landforms and ecological systems. In this ecological system, the wild rodents of *Apodemus chevrieri* and *Eothenomys miletus* are the main reservoir hosts, and the fleas of *Neopsylla specialis* and *Ctenophthalmus quadratus* are the main vectors [4, 9]. The major rodent hosts in these two plague foci are the same wild rodents, which differ completely from the domestic rodents such as *Rattus flavipectus*, etc., that are found in local residents' houses. The two plague foci were coined wild rodent (sylvatic) plague foci by Chinese plague researchers [9].

In this study, the epizootics in the Yulong focus were described, and three molecular subtyping methods, the different region (DFR) analysis, clustered regularly interspaced short palindromic repeats (CRISPRs), and the multiple-locus variable number of tandem repeat (VNTR) analysis (MLVA) (14+12), were used to genotype and source-trace the *Y*. *pestis* isolates in the Yulong plague focus. Simultaneously, several isolates from the vicinity of Yunnan were used as controls.

## Methods

### Ethics statement

All procedures performed in this study were in accordance with the ethical standards of the institutional and national research committee. This study was approved by the Review Board of Ethics in the National Institute for Communicable Disease Control and Prevention, China CDC. The review board approved the collection and use of rodents in this study.

### Surveillance of the Yulong plague focus

During 2006-2016, 2 surveillance periods were conducted annually, one in the spring (April to May) and one in autumn (November to December), for approximately 15 days each time. Plague surveillance was concentrated within 30 kilometers of the center of Luzi village; this area included 25 villages in 2 townships in Yulong County and 9 villages in 1 township in Gucheng District. The collection methods included live rat capturing and dead rat searching in the surrounding villages, farmland and woodland. Captured rodents and dead rodents were sent to the laboratory and analyzed.

### Identification of sylvatic plagues in Yulong focus

The confirming tests of animal plagues were performed according to the World Health Organization’s criteria and the animal plague surveillance criteria issued by the China CDC (2008). These assays included bacterium isolation, PCR tests and immunoassays (F1 antigen test by RIHA). The total DNA of dead rodents was extracted according to DNeasy Blood & Tissue Kit (QIAGEN) instructions, and these DNAs served as templates for the PCR test (real-time PCR and common PCR kits, Shanghai Huirui Biotechnology Co., Ltd.).

### Strains and genomic DNA

A total of 46 *Y*. *pestis* isolates were collected from three natural plague foci in Yunnan province and its surrounding areas in this study (S1 Table). Fourteen strains of the *R*. *flavipectus* plague focus (including 2 in Burma, 2 in Guangxi province, 2 in Guizhou province, and 9 in Yunnan province), 8 strains of the Jianchuan plague focus, 6 strains from Tibet, 5 strains from Sichuan province, and 7 strains from the Yulong plague focus with an additional 5 *Y*. *pestis* DNA templates were obtained from the Yulong plague focus in 2014. The bacterial genomic DNAs were extracted by conventional SDS lysis and phenol-chloroform extraction methods [5].

### Molecular subtyping analysis

DFR genotyping and CRISPR analyses were performed according to previous reports [6–8, 11, 12]. Twenty-three DFR primers and *pMT*1-specific primers were used to identify DFR loci. The spacer arrays of CRISPRs were gained in ‘‘spacers dictionary’’ [6] or analyzed online using the ‘‘CRISPR Finder Tool’’ in the CRISPRs database [13]. The nomenclature of genotypes in the DFR and CRISPR analysis were employed according to previous studies [6, 7]. The profile data of DFR and CRISPRs were compared using Bionumerics 6.6 (Applied Math), and the corresponding MST (minimum spanning tree) was drawn for the cluster analysis. If there were differences at only 1 locus between 2 neighboring types, they would be surrounded by a halo of the same color and form a complex. The strains in one complex of Lijiang strains were used for the next tracing analysis by MLVA.

### Source tracing analysis

The MLVA analysis with 26 markers (14+12) was performed as described by Li et al [5] with the following modifications on capillary electrophoresis. The forward primers were labeled with different fluorescent dyes, FAM or Hex. The PCR amplification was diluted with water to 1:80. After denaturing by heating, the amplicons were separated by capillary electrophoresis on an ABI 3730xl genetic analyzer with a GeneScan 1200 LIZ size standard (Applied Biosystems). The lengths of the amplicons were determined according to the sizes generated by GeneMapper software V. 4.0 (Applied Biosystems).

The profile data of MLVA (14+12) were compared using Bionumerics 6.6 (Applied Math). In addition to the VNTR data in our 23 *Y*. *pestis* isolates (S2 Table), an additional 83 representative strains from previous MLVA (14+12) studies were also included for the cluster analysis[5] (S3 Table). The genotyping criteria and naming refers to the paper of Cui et al [3]. The MLVA profiles were analyzed as a characteristic data using the alignment of the categorical coefficient and UPGMA (unweighted pair group method using arithmetic averages). The dendrogram was constructed using the minimum spanning tree (MST) by parameters (maximum and minimum neighbor distances were all selected as 1).

## Results

### Animal plague epidemics in the Yulong plague focus, 2006-2016

A total of 6937 live wild rodents were captured. The rodents comprised 22 species, of which 51.66% were *Apodemus chevrieri*, 20.91% were *Eothenomys miletus*, and 7.88% were *Eothenomys proditor*. A total of 75 dead rodents were obtained. Additionally, 1323 fleas were isolated from rodents. The fleas comprised 12 species, of which 51.46% were *Neopsylla specialis*, 24.43% were *Ctenophthalmus quadratus*, and 12.82% were *Frontopsylla spadix*. For all live rodents and their fleas, the bacteria isolation, RIHA and specific PCR results were negative for *Y*. *pestis*. However, 14 of dead rodents tested positive for *Y*. *pestis*, as did 2 fleas from dead rodents (positive dead rodents).

After the Yulong plague focus was identified by bacteriological evidence in 2006, continuous rodent plague epidemics were identified in the main plague reservoirs. During the 10-year period of 2006-2016, animal plague epidemics occurred in 6 years. As a central area, cases were frequently reported in the Luzi village during these 6 years. In 2008, the Mangzhong Village, which is located approximately 8 km northwest of the Luzi village, experienced an animal plague epidemic. A total of seven *Y*. *pestis* stains were isolated in 2006, 2008 and 2009, and sixteen animal samples were positive for RIHA in 2006-2009, 2012 and 2014 (Table 1). Notably, the rodent plague occurred in 2014. In addition to the positive results of the five dead mice based on the RIHA test (four of *Apodemus chevrieri* and one of *Eothenomys miletus*), DNA templates extracted from the five dead mice were also positive according to *Y*. *pestis* specific gene PCR (*caf1* and YPO0392). However, no strain was successfully isolated from these mice because of their rotted bodies. The 5 villages of Luzi, Mangshang, Mushu, Mangzhong and Runanhua have all undergone animal plague epidemics in a 30-km^2^ area. (Fig 1) This evidence suggests that continuous epidemics of rodent plague have existed in the Yulong plague focus since 2005.

**Table 1:** Rodent plague occurring in the Yulong plague focus, 2006-2016.

Year	Suffered village	Specimen code	Bacteria isolation	RIHA	Specific PCR
2006	Luzi	2006-3	+	+	+
		2006-4	+	+	+
		2006-7	+	+	+
		2006-5	+	+	+
		2006-8	+	+	+
2007	Luzi	2007-1	-	+	-
2008	Luzi	2008-1	-	+	-
		2008-3	+	+	+
	Mangzhong	2008-2	-	+	-
2009	Luzi	2009-1	+	+	+
2010-2011	none				
2012	Luzi	2012-1	-	+	-
2013	none				
2014	Luzi	2014-3	-	+	+
	Mangzhong	2014-5	-	+	+
	Mushu	2014-7	-	+	+
	Mangshang	2014-2	-	+	+
	Runanhua	2014-1	-	+	+
2015	none				
2016	none				

### Subtyping analysis of DFR and CRISPRs

**Fig 2:**
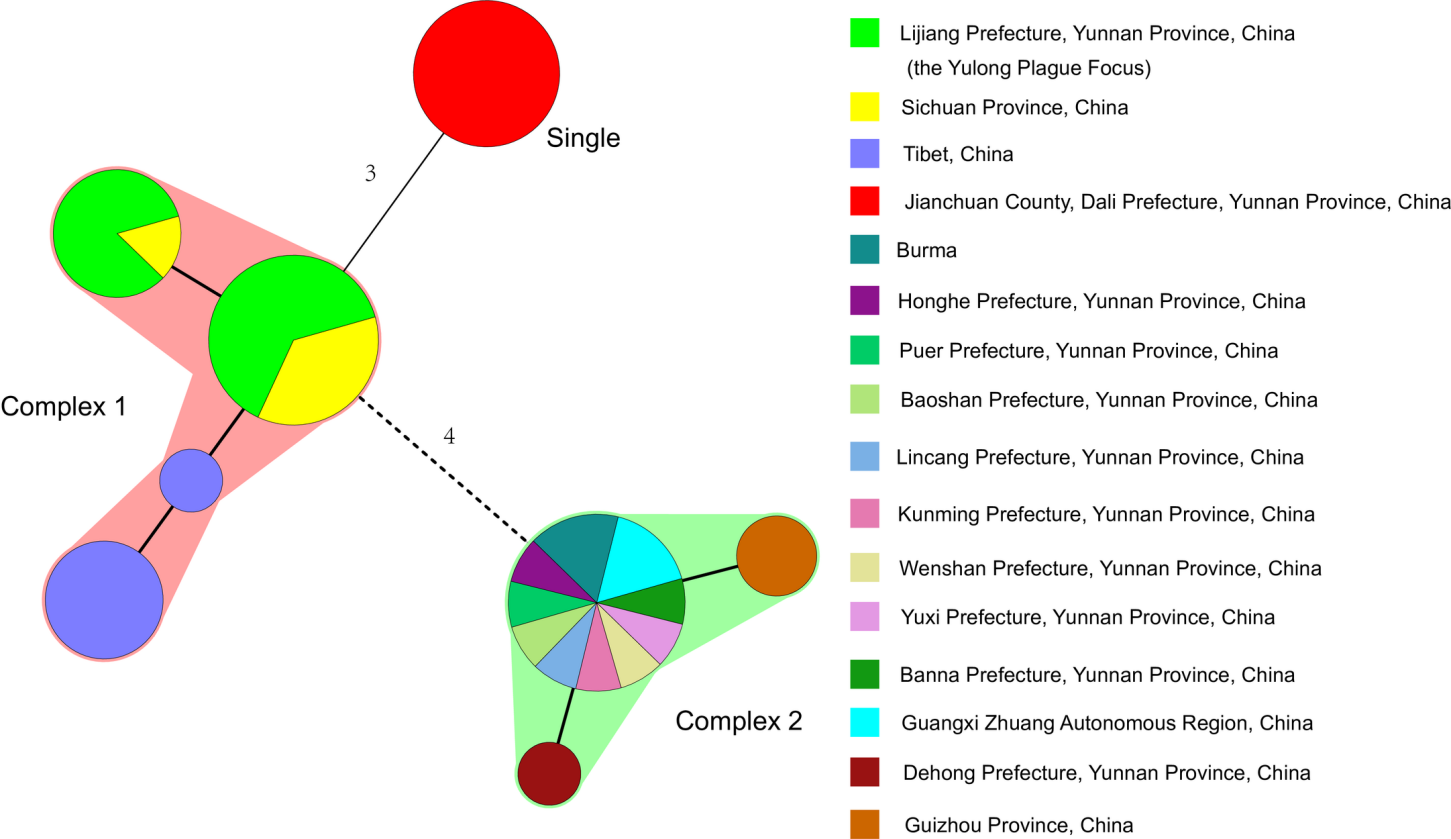
Minimum spanning tree analysis of DFR & CRISPR to *Yersinia pestis* strains in the Yulong plague focus and its surrounding areas. A minimum spanning tree was constructed using the DFR and CRISPR genotyping data (S1 Table). The DFR and CRISPR types are displayed as circles, and the size of the circle indicates the number of isolates with the particular type. Thick solid lines connect types that differ in a single locus, thin solid lines connect types that differ in 3 loci, and dashed lines connect types that differ in 4 loci. If 2 neighboring types do not differ in more than 1 locus, they are surrounded by a halo of the same color and form a complex.

The 23 indexes of DFR, and the 16 indexes of CRISPR, were used to cluster the 47 strains of *Y*. *pestis* in this study (S1 Table). Based on a complex definition of no more than 1 mutation of adjacent distance, the strains of Lijiang, Sichuan and Tibet were of the same complex (Fig 2, Complex 1), and all 14 strains of the *R*. *flavipectus* plague focus were another complex (Fig 2, Complex 2). All 8 strains of the Jianchuan plague focus were uniquely different from Complex 1 and Complex 2 (Fig 2, Single). In complex 1, a further analysis is necessary to study what relationship exists among the Lijiang, Sichuan and Tibet strains. The DFR genomovars of seven isolations and five positive *Y*. *pestis* DNAs in the Yulong plague focus were identified as genomovar 05 in this study [7], as were the strains of Sichuan and Tibet (Fig 2 and S1 Table). The DFR genomovar of *Y*. *pestis* in the Jianchuan plague focus was identified as genomovar 07, whereas the DFR of the *R*. *flavipectus* plague focus was identified as genomovar 09 [7].

The CRISPR patterns of seven isolations and five positive *Y*. *pestis* DNAs in Yulong was identified as genotype 22 in the Ca7 cluster, i.e., Ypa (a1-a2-a3-a4-a5-a6-a7), Ypb (b1-b2-b3-b4), and Ypc (c1-c2-c3), whereas the arrays of spacers in the Jianchuan focus were genotype 35 in the Ca52 cluster [6], and the spacer arrays of CRISPRs in the *R*. *flavipectus* plague focus were genotypes 30 or 33 in the Ca8 cluster [6] (S1 Table). The CRISPR patterns of the Yulong plague focus were also found in other plague natural focuses such as *Y*. *pestis* isolates in Sichuan province in 2009 and 2011 and in Tibet in 1978 and 2011 (S1 Table).

### The genetic relationship of *Y*. *pestis* illustrated by MLVA “14+12”

**Fig 3:**
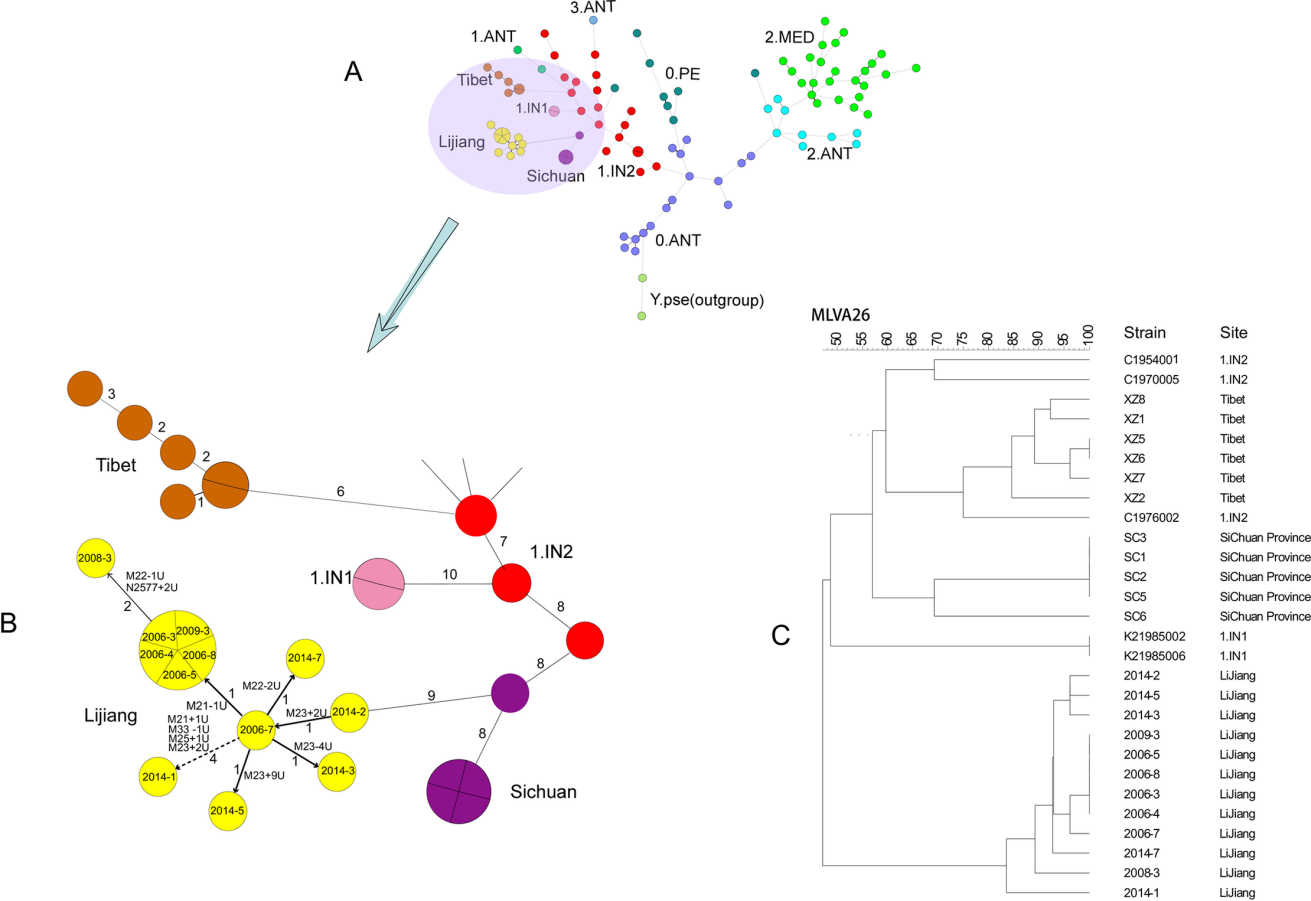
Minimum spanning tree analysis of the MLVA “14+12” scheme to Yulong *Yersinia pesti*s strains, Sichuan strains, Tibet strains, 81 representative strains and 2 of *Y*. *pseudotuberculosis* strains. A: Minimum spanning tree of all 106 strains involved in our study. B: Minimum spanning tree of Yulong, Sichuan and Tibet strains. C: An MLVA dendrogram of Yulong, Sichuan, and Tibet strains.

The MLVA (14+12) scheme was used for the phylogenic structure analysis and for the source-tracing investigation; it was considered to produce a mostly approximated phylogenic structure and relationship with the SNP-based analysis[5]. There were 18 discrepant VNTR loci in the Yulong, Sichuan and Tibet isolates (Table 2). The genetic relationship of the VNTR profiles of the strains in this study with profiles in previous studies [5] is illustrated in the MST tree (Fig 3 and S2 Table). The tree shows that the Tibet and Sichuan strains evolved from strains 1.IN2 (Qinghai, 1970 and Tibet, 1976), and the Lijiang strains are from a clone of the Batang plague focus in Sichuan province (Batang County, 2011). The Batang plague focus in Sichuan province is located to the north approximately 350 km away from the Yulong plague focus. Within Lijiang strains, one strain (2014-2) was the earliest clone isolated in 2014 from Mangshang village, which is also the northernmost part of the Yulong plague focus. This strain then spread to Luzi Village and formed a new clone (2006-7) by adding 2U repeats in the M23 site. The clone of 2006-7 continues to spread around, producing new clones through mutations and creating new animal plague epidemics. Notably, a significant mutation (4 loci) occurred during the transmission of *Y*. *pestis* clones from Luzi village to Runahua village. Topographically, the distance between the two villages is approximately 6 km, but there is a mountain barrier that forms a natural barrier, whereas there is no natural barrier among the Luzi, Mangzhong and Mushu villages.

**Table 2:** The profiles of discrepant VNTR of *Y*. *pestis* in the MLVA “14+12” scheme in three plague foci in Yunnan and other plague foci in China.

Strain ID	Repeat Numbers																	
	M58	M21	M15	M61	N2486	N3779	N2117	N1606	N2577	N3773	M33	M34	M22	M43	M25	M23	M28	M29
2006-5	7	3	5	4	3	4	6	6	10	4	20	3	21	5	17	13	3	7
2006-3	7	3	5	4	3	4	6	6	10	4	20	3	21	5	17	13	3	7
2006-8	7	3	5	4	3	4	6	6	10	4	20	3	21	5	17	13	3	7
2006-4	7	3	5	4	3	4	6	6	10	4	20	3	21	5	17	13	3	7
2006-7	7	4	5	4	3	4	6	6	10	4	20	3	21	5	17	13	3	7
2008-3	7	3	5	4	3	4	6	6	12	4	20	3	20	5	17	13	3	7
2009-3	7	3	5	4	3	4	6	6	10	4	20	3	21	5	17	13	3	7
2014-1	7	5	5	4	3	4	6	6	10	4	19	3	21	5	18	15	3	7
2014-2	7	4	5	4	3	4	6	6	10	4	20	3	21	5	17	11	3	7
2014-3	7	4	5	4	3	4	6	6	10	4	20	3	21	5	17	9	3	7
2014-5	7	4	5	4	3	4	6	6	10	4	20	3	21	5	17	22	3	7
2014-7	7	4	5	4	3	4	6	6	10	4	20	3	19	5	17	13	3	7
XZ1	8	4	5	3	3	4	10	5	9	5	19	9	18	4	15	7	6	7
XZ2	8	4	5	3	3	4	10	7	9	5	19	8	17	4	15	7	6	7
XZ5	8	4	3	3	3	4	10	8	9	5	19	10	18	4	15	7	6	7
XZ6	8	4	3	3	3	4	10	8	9	5	19	10	18	4	15	7	6	7
XZ7	8	4	3	3	3	4	11	8	9	5	19	10	18	4	15	7	6	7
XZ8	8	4	3	3	3	4	10	9	9	5	19	9	18	4	15	7	6	7
SC1	8	4	5	4	6	4	9	7	9	5	20	8	17	5	16	6	6	6
SC2	8	4	5	4	6	4	9	7	9	5	20	8	17	5	16	6	6	6
SC3	8	4	5	4	6	4	9	7	9	5	20	8	17	5	16	6	6	6
SC5	8	4	5	4	6	4	9	7	9	5	20	8	17	5	16	6	6	6
SC6	8	4	5	4	3	3	6	8	8	5	20	8	18	5	17	11	6	6

## Discussion

Plague is an historical and continuous problem in many rural regions in China. The evidence of bacterium isolation and immunoassays in local reservoirs indicates that continuous rodent plague has been prevalent in the Yulong plague focus since the focus was discovered in 2005. In our study, although no *Y*. *pestis* strain was successfully isolated in the dead rodents in 2014, we still successfully used the total DNA samples of dead rodents as materials to perform molecular subtyping. Therefore, clinical tissue obtained from humans or specimens from rodents can also be used in PCR-based molecular genotyping. This practice can be useful in microbial forensic investigations, such as in human plague outbreaks or bioterrorism attacks.

Different molecular subtyping methods are used for different purposes. With the advantages of lower cost and more feasibility, DFR, CRISPRs and the MLVA (14+12) method, together with the corresponding database [14], could provide a feasible tool for source-tracking investigation [5–7, 15, 16]. CRISPR and DFR analyses were previously used to illustrate the phylogenetic relationship and microevolution of *Y*. *pestis* in China[5–8]. *Y*. *pestis* isolated from the Yulong or Jianchuan foci belonged to the Biovar *Antique* [12], whereas strains in the *R*. *flavipectus* plague focus were from the Biovar *Orientalis* [7].

The genomovar 05 of DFR was previously identified in the *Marmota himalayana* plague focus of the Qinghai–Gansu–Tibet Grassland (Focus C) and the *Marmota himalayana* plague focus of the Kunlun Mountains (Focus K2) [7]. The difference between the Yulong and *R*. *flavipectus* plague focus was that the Yulong plague focus lacked DFR13, which encodes a filamentous prophage integrated into the chromosomal *dif* locus [7], whereas the strains in the *R*. *flavipectus* plague focus lack DFR3. One interesting observation was the difference of genotypes in DFR between the Yulong focus and the Jianchuan focus. Although the two foci are adjacent to one another, the landforms and their main reservoirs are similar. However, the DFR genomovar of *Y*. *pestis* in the two foci was different. The *Y*. *pestis* of the Jianchuan focus possessed the DFR4 locus, with the corresponding functions annotated as adherence proteins [11].

The CRISPR patterns of *Y*. *pestis* isolates in the Yulong focus were identified as genotype 22 in the Ca7 cluster; these results were consistent with previous reports [9]. In addition, this CRISPR pattern was also identified in the *Marmota caudate* plague focus of the Pamirs Plateau (Focus A), the *Marmota baibacina–Spermophilus undulates* plague focus of the Tianshan Mountains (Focus B), the *Marmota himalayana* plague focus of the Qinghai–Gansu–Tibet Grassland (Focus C), the *Marmota himalayana* plague focus of the Kunlun Mountains (Focus K), and the *Marmota focus* plague focus of the Qinghai–Tibet Plateau (Focus M) [6].

Some MLVA schemes, such as 25 or 42-46 VNTR markers, were used to illustrate the phylogenetic relationships of *Y*. *pestis* [5, 14, 15, 17–20]. In our previous research, a scheme including 14 VNTR loci was performed to analyze a total of 213 Chinese *Y*. *pestis* strains, which included five strains isolated from the Yulong Plague focus in 2006 [4]. Common gel electrophoresis was used to identify the size of the PCR products [4]. Therefore, only VNTR loci with conservative tandem repeat sequences above 9 bps were selected as MLVA profiles from previously described VNTR loci [18]. Those strains (n = 5) of the Yulong focus involved in this study [4] presented different MLVA types (MT17 types) with other natural plague foci in China. The cluster analysis in this study also suggested that the Yulong strains show a closer genetic relationship with the strains from the *Marmota himalayana* plague focus of the Qinghai-Gansu-Tibet Grassland (Focus C) than the *Apodemus chevrieri* and *Eothenomys miletus* plague foci of the Jianchuan plague focus (Focus E) [4]. It should be mentioned that, after our previous research about “14-above 9 bp -repeats” MLVA schemes, other MLVA schemes were developed by serial hierarchical assessment, and the sizes of the PCR products were resolved by capillary electrophoresis [7], such as the MLVA “14+12” scheme. Compared to the VNTR loci selected in the scheme MLVA “14-above 9 bp -repeats” [4] mentioned above, only two VNTR foci (M61 and M58) were involved in the scheme MLVA “14+12”.

In this study, our research performed the MLVA “14+12” scheme to analyze the phylogenetic relationship of *Y*. *pestis* in three plague foci in Yunnan province and other plague foci in China in available previous studies [5]. We reasoned that the MLVA”14+12” scheme had the ability to obtain a phylogeny relationship mostly approximate to the SNP-based analysis[5] and possessed high discriminative ability in genotyping and could be used for source tracing.

The question of where the Yulong plague focus comes from has been asked since it was confirmed in 2006. This study shows that the Yulong strains originated from the Sichuan Batang strains of Himalaya marmot plague foci, which is consistent with the plague spreading in a route from the north to the south in China, as previously described by Morelli G [2]. The Luzi village is located in the center of the focus and was the first discovered plague epidemic; it also had the highest frequency of infection in the epidemic area. However, the tracing results of MLVA (14+12) showed that the strains from Mangshang Village were the earliest strains. The Mangshang village is located in the northernmost part of the Yulong plague focus, and the transmission line of *Y*. *pestis* in the Yulong focus also goes from the north to the south, similar to the plague spreading route in China.

We observed the phenomenon that the profiles of MLVA (14+12) in the DNA from the five *Y*. *pestis* strains collected in 2014 are not completely consistent (Fig 3 and S3 Table). In the Yulong plague focus, the geographic landscape consists of woods separated by cultured farm, which forms separated micro-foci. The above observation suggests that the habitat segregation of main reservoirs could cause a few phylogenetic differences in *Y*. *pestis* in the plague focus.

In conclusion, the 10-year monitoring period showed that the plague epidemic continued to exist and expand among the host rodents in the Yulong plague focus. Searching for dead mice was the most effective monitoring method in this plague focus. The plague information has not been detected in the captured live rodents (nearly 7000) thus far, suggesting that the virulence of strains in the Yulong plague focus is stronger and the survival time of mice is shorter after infection. In terms of genetic or phylogenic relationships, Lijiang strains are closer to Batang strains of the Himalaya marmot plague foci. In summary, we have obtained a deeper understanding of this new plague focus through this study, which provides a basis for effective prevention and control. Moreover, we also provide a set of paradigms for the systematic study of new plague foci, which is a perfect combination of traditional monitoring methods and modern research methods.

## Supporting information

S1 Table Information and CRISPR & DFR loci in 46 *Y*. *pestis* strains. (XLSX)

S2 Table The profiles of 26 VNTR of *Y*. *pestis* in the MLVA “14+12” scheme in three plague foci in Yunnan and other plague foci in China. (XLSX)

S3 Table The profiles of 26 VNTR in the MLVA “14+12” scheme of 81 representative *Y*. *pestis* and 2 *Y*. *pseudotuberculosis* strains. (XLSX)
